# Approximate and Exact Optimization Algorithms for the Beltway and Turnpike Problems with Duplicated, Missing, Partially Labeled, and Uncertain Measurements

**DOI:** 10.1089/cmb.2024.0661

**Published:** 2024-10-30

**Authors:** C.S. Elder, Minh Hoang, Mohsen Ferdosi, Carl Kingsford

**Affiliations:** ^1^ Ray and Stephanie Lane Computational Biology Department, Carnegie Mellon University, Pittsburgh Pennsylvania, USA.; ^2^ Lewis-Sigler Institute for Integrative Genomics, Princeton University, Princeton, New Jersey, USA.

**Keywords:** Inverse Problem, Distance Problem, Optimization, Beltway, Turnpike

## Abstract

The Turnpike problem aims to reconstruct a set of one-dimensional points from their unordered pairwise distances. Turnpike arises in biological applications such as molecular structure determination, genomic sequencing, tandem mass spectrometry, and molecular error-correcting codes. Under noisy observation of the distances, the Turnpike problem is NP-hard and can take exponential time and space to solve when using traditional algorithms. To address this, we reframe the noisy Turnpike problem through the lens of optimization, seeking to simultaneously find the unknown point set and a permutation that maximizes similarity to the input distances. Our core contribution is a suite of algorithms that robustly solve this new objective. This includes a bilevel optimization framework that can efficiently solve Turnpike instances with up to 100,000 points. We show that this framework can be extended to scenarios with domain-specific constraints that include duplicated, missing, and partially labeled distances. Using these, we also extend our algorithms to work for points distributed on a circle (the Beltway problem). For small-scale applications that require global optimality, we formulate an integer linear program (ILP) that (i) accepts an objective from a generic family of convex functions and (ii) uses an extended formulation to reduce the number of binary variables. On synthetic and real partial digest data, our bilevel algorithms achieved state-of-the-art scalability across challenging scenarios with performance that matches or exceeds competing baselines. On small-scale instances, our ILP efficiently recovered ground-truth assignments and produced reconstructions that match or exceed our alternating algorithms. Our implementations are available at https://github.com/Kingsford-Group/turnpikesolvermm.

## 1. INTRODUCTION

The Turnpike problem is a classical algorithmic challenge that arises in several biological domains. Given the unordered set of pairwise distances among *n* unknown points on a line, the goal of Turnpike is to reconstruct the locations of the points. A well-known variant of Turnpike, the Beltway problem replaces the requirement that the points are on a line with the constraint that the points lie on a circle. Applications of Turnpike and Beltway include tandem mass spectrometry, biomolecule structure estimation (Huang and Dokmanić, 2021), de novo sequencing of linear and cyclic peptides (Mohimani et al., 2011; Fomin, 2015), and reconstruction of DNA sequences from their partially digested fragments (Smith and Birnstiel, 1976; Skiena and Sundaram, 1993). Variants of the Beltway and Turnpike problems are also applied to quantum phase estimation (Zintchenko and Wiebe, 2016), molecular error-correcting codes used for databases (Gabrys et al., 2020), and generalized orthogonal measurements (Bendory et al., 2024).

When all pairwise distances are observed without error, Turnpike can be solved with a backtracking algorithm that has exponential runtime on rare worst cases (Zhang, 1994) and an expected runtime of $O(n^{2}\;\log\;n)$ on random instances (Skiena et al., 1990; Skiena and Sundaram, 1993). In contrast, algorithms for Beltway are less efficient, with a worst-case runtime of $O(n^{n}\;\log\;n)$ that is often realized in practice (Fomin, 2015). Various algorithms have been proposed to improve both empirical (Abbas and Bahig, 2016; Lemke et al., 2003) and worst case run time (Nadimi et al., 2011) of Turnpike and Beltway. However, these approaches are highly susceptible to numerical precision errors and take more time than the backtracking algorithm in practice.

Observed distances are usually noisy due to uncertainty in the measuring equipment (Huang and Dokmanić, 2021). This leads to the Noisy Turnpike and Noisy Beltway variants, which are both strongly NP-complete (Cieliebak and Eidenbenz, 2004). The backtracking approach can be modified to use intervals instead of points to accommodate measurement uncertainty (Skiena and Sundaram, 1993), but these modifications lead to the consideration of exponentially many paths, limiting the algorithm’s efficiency and practical applicability (Huang and Dokmanić, 2021). Modifications can be made to solve Noisy Beltway by eliminating redundant measurements, but as in the exact case, only very small Noisy Beltway instances can be solved with this algorithm (Fomin, 2019). In the special case of partial digestion of DNA, Pandurangan and Ramesh (2002) use the additional assumption that distances from both ends of the double-stranded DNA sample are observed. More recently, Huang and Dokmanić (2021) model both the Noisy Turnpike and Noisy Beltway problems as probabilistic inference of point assignments using discrete bins that quantize the input domain. This approach assumes that no bin can contain more than one point, which only holds when the magnitude of the observation noise is sufficiently small relative to the smallest distance. As such, the accuracy of this algorithm tends to deteriorate in noisier instances. It also struggles to efficiently solve larger problem instances, as shown in our empirical study.

We propose a new approach that casts the problems Noisy Turnpike and Noisy Beltway as joint optimization over (a) the unknown point set and (b) a permutation ordering the distances by magnitude. This reformulation, which is detailed in Section 2, results in algorithms that efficiently solve large, noisy instances of Turnpike and Beltway. Our first contribution is a bilevel optimization scheme that alternates between estimating the point-to-distance matching and recovering the point set with this assignment. Our formulation’s non-convex optimization landscape contains many saddle points and local optima. We accommodate for this by introducing a divide-and-conquer step to recursively correct common small-scale mistakes that lead to low-quality solutions. Our algorithm runs in time $O(n^{2}\;\log\;n)$ for each step, with time dominated by a low-cost sorting step. Our second contribution is a general purpose integer linear program (ILP) that finds globally optimal reconstructions for *support function* objectives, which are a generic family of convex functions including norms. For this formulation, we construct an extended formulation modeled after one found in Goemans (2015) that reduces the number of binary variables from $O(n^{4})$ to $O(n^{2}\;\log\;n)$. In Section 3, we formulate three problem-specific objectives in this form and then test them in difficult settings at various scales. In our tests, the ILP formulation efficiently solved experiments of moderate size ( ∼30 or fewer points) to global optimality, but the formulation struggled with sizes exceeding this threshold. Thus, the ILP is best for moderately-sized applications that require optimal reconstructions.

We empirically demonstrate the performance of our proposed bilevel algorithm and subsequently show that it outperforms state-of-the-art methods in various synthetic and realistic biological settings, such as the partial DNA digestion task (Huang and Dokmanić, 2021). Our algorithm comes to accurate solutions even under extremely noisy observation conditions. We also demonstrate that the proposed algorithm runs more efficiently than previous approaches, capable of handling partial digestion instances with up to a hundred thousand digested fragments. This means that our algorithm scales to genome-sized applications, which is not possible with previous methods. We additionally extend our algorithm to a generalized one-dimensional distance matching problem that accepts partitioned and partially labeled distances along with a set of known missing distances. In summary, our algorithms advance the capacity to address the Noisy Turnpike Problem, the Noisy Beltway Problem, and general one-dimensional distance matching problems in biological and general contexts in a way that provides high accuracy and scalability.

## 2. METHOD

### 2.1. Problem setting

Let m=n(n−1)/2 and $D\in {\mathbb{R}}^{m}$ be a vector of pairwise distances between *n* points. We denote the ground truth vector that contains these points as $z\in {\mathbb{R}}^{n}$. Without loss of generality, we assume that $z_{1}\leq \ldots \leq z_{n}$, $\sum_{k=1}^{n}z_{k}=0$, and $∥z{∥}_{2}=1$; that is, the unknown points are named in sorted order, centered around zero, and have unit norm. These assumptions do not fundamentally change the problem but are important nonetheless, as they prevent trivial non-uniqueness. The first and second assumptions are valid because the distance set is invariant to the translation and permutation of the points, allowing us to search for a centered and sorted solution vector *z*. The third assumption follows because we can construct a set of scaled distances $\bar{D}=\sqrt{n}\;∥D{∥}_{2}^{-1}\;D$ from the original distances. This rescaling generates $\bar{z}=z/∥z{∥}_{2}$ because

$$\|D{\|}_{2}^{2}=\sum_{i\;⩽\;j}^{n}{\left(z_{j}-z_{i}\right)}^{2}=n\|z{\|}_{2}^{2}+\sum_{ij}^{n}z_{i}z_{j}=n\|z{\|}_{2}^{2}+{\left(\sum_{k=0}^{n}z_{k}\right)}^{2}=n\|z{\|}_{2}^{2},$$

where we used the fact that *z* is centered.

Let Z be the set of vectors in ${\mathbb{R}}^{n}$ that satisfy the above assumptions (considering the dimensions of the vectors in Z as the point locations), and let $S_{m}$ denote the set of all (m×m) permutation matrices. When statements apply to both, we refer to the variants Exact Turnpike and Noisy Turnpike as Turnpike without distinction. The Exact Turnpike problem is formalized as finding a vector $\hat{z}\in Z$ such that $Q\hat{z}=PD$ for some $P\in S_{m}$, and where $Q\in {\mathbb{R}}^{m\times n}$ is a fixed incidence matrix defined as follows. Each row in *Q* corresponds to a pair of indices i<j. For convenience, we let the function α(i,j) map the index pair (i,j) to its (arbitrary) row index in *Q*. The incidence matrix *Q* is constructed such that $Q_{\alpha (i,\;j),\;j}=1$ and $Q_{\alpha (i,\;j),\;i}=-1$ are the only non-zero entries in $Q_{\alpha (i,\;j)}$. It follows that $Q_{\alpha (i,\;j)}\hat{z}={\hat{z}}_{j}-{\hat{z}}_{i}$, and $Q\hat{z}$ contains the complete pairwise distance collection generated by $\hat{z}$. Furthermore, if $\hat{z}$ recovers the ground truth *z*, then $Q\hat{z}$ must also be a permutation of *D*, which explains the role of *P* in the objective. In the Noisy Turnpike case—which is the focus of this paper—exact recovery is generally not possible due to the corrupted observations. To approximate exact recovery, we formulate an optimization task to regularize potential solutions:

(1) $$\hat{z}=\underset{z^{\prime }\;\in \;\;Z}{\text{argmax}}\;\underset{P\in S_{m}}{\text{max}}\;\langle Qz^{\prime },PD\rangle ,$$

where 〈·,·〉 denotes the inner product of two vectors. This is equivalent to minimizing the $\ell_{2}$ distance between $Qz^{\prime }$ and *PD* because the norm of *P, Q* and *D* are constant, $z^{\prime }$ is normalized based on our previous assumptions, and optimality in the exact case will take place when $Qz^{\prime }=PD$. However, Eq. (1) is more convenient for our subsequent derivation.

### 2.2. Minorization-Maximization scheme for optimizing the Turnpike objective

**Algorithm 1:**   Minorization-Maximization (MM)**Input:**   Distance vector *D*, initial estimate $z^{\left(0\right)}$, tolerance ϵ>0  1:  t←02: $\;D\leftarrow D^{↑}$ ▷ Replace *D* with its sorted equivalent $D^{↑}$3:   **while not** converged **do**4: $P_{t}\leftarrow \Pi_{Qz^{\left(t\right)}}^{⊤}$ ▷ Calculate and sort $Qz^{\left(t\right)}$ using Alg. 25: $z^{\left(t+1\right)}\leftarrow Q^{⊤}P_{t}D$ ▷ Estimate the next point vector6: t←t+17:   converged $\leftarrow ∥z^{\left(t+1\right)}-z^{\left(t\right)}{∥}_{2}<\epsilon$8:   **end while**9:   **return** $\text{unit}(z^{\left(t\right)})$

Eq. (1) is a bilinear program, as fixing either *P* or *z* reduces the problem to a linear program in the remaining variable. This motivates a bilevel MM scheme (Sun et al., 2017) to optimize this objective. We do this by relaxing our objective into two alternating subproblems. At iteration t+1, the first subproblem fixes a surrogate point vector $z^{\left(t\right)}$ and solves for

(2) $$P_{t}=\underset{P\;\;\in \;\;S_{m}}{\text{argmax}}\;\langle Qz^{\left(t\right)},PD\rangle ,$$

which has a closed-form solution, as shown in Proposition 1. This closed form is a variant of the rearrangement inequality (Hardy et al., 1952), a connection highlighted through Lemma 1.

**Lemma 1.** Suppose $y=(y_{1},y_{2},\ldots ,y_{n})\;\in \;{\mathbb{R}}^{n}$ is a sorted vector, i.e., $y_{1}\leq y_{2}\leq \ldots \leq y_{n}$ holds. The problem

(3) $$\underset{P\;\in \;S_{m}}{\text{argmax}}\;\langle Px,y\rangle$$

is maximized by any permutation $P_{x}$ such that $P_{x}x$ is in sorted order.

**Proof:** Let $P\in S_{m}$ be a non-sorting permutation, which means there exist indices i<j such that ${\left(Px\right)}_{i}>{\left(Px\right)}_{j}$. Then transposing elements *i* and *j* increases the objective because

$$({\left(Px\right)}_{j}-{\left(Px\right)}_{i})(y_{j}-y_{i})\leq 0\Rightarrow {\left(Px\right)}_{j}y_{j}+{\left(Px\right)}_{i}y_{i}\leq {\left(Px\right)}_{i}y_{j}+{\left(Px\right)}_{j}y_{i}.$$

This implies the permutation that applies *P* then transposes *i* and *j* is no worse than *P*. Iterating this argument leads to a sorting permutation that is also no worse than *P*. The initial permutation was arbitrary, so there exists an optimal permutation that sorts *x*. Finally, note that any two sorting permutations *P* and $P^{\prime }$ have the same objective value since sortedness implies $Px=P^{\prime }x$.□

**Proposition 1.** Let $\Pi^{⊤}$ be a permutation that puts *Qz* into sorted order. The permutation Π is a globally maximizing solution to Eq. (2).

**Proof:** Without loss of generality, we assume that *D* is sorted as a preprocessing step to the Noisy Turnpike problem. Eq. (2) then transforms into $\langle Qz^{\left(t\right)},PD\rangle =\langle P^{⊤}(Qz^{\left(t\right)}),D\rangle$. By Lemma 1, a permutation $\Pi^{⊤}$ that sorts *Qz* must be a global maximizer of the right-hand side. Notice the transpose Π is the equivalent solution on the left-hand side, proving the claim.□

On the other hand, the second subproblem fixes an estimation for $P_{t}$, which is the closed-form solution derived above. This subproblem then solves for

(4) $$z^{\left(t+1\right)}=\underset{\hat{z}\;\in \;Z}{\text{argmax}}\;\langle Q\hat{z},P_{t}D\rangle \;\equiv \underset{\hat{z}\;\in \;Z}{\text{argmax}}\langle \hat{z},Q^{⊤}P_{t}D\rangle \;.$$

Since the inner product of two vectors is maximized when they are parallel and $∥\hat{z}{∥}_{2}=1$ by assumption, the maximum objective value is obtained when $\hat{z}=\text{unit}(Q^{⊤}P_{t}D)$, where unit(·) scales a vector to unit norm.

**FIG. 1.**
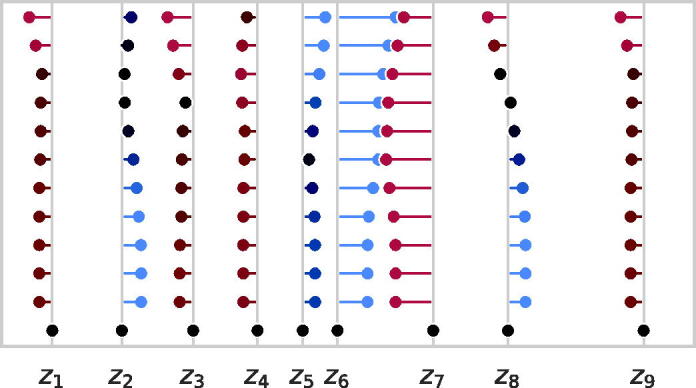
A run of Alg. 1 on a 9-point distance set where the gray lines emanating from the x-axis denote ground truth points. Estimated points for each iteration are drawn from top (the initializer, iteration zero) to the bottom (final iteration, the solution), and an additional line is drawn from each estimated point to the closest ground truth point. These lines are colored red, black, or blue to indicate that the estimated point is too far to the left, nearly matched, or too far to the right relative to the respective ground truth point. Note that, by the final iteration, the estimated points are perfectly matched to the ground truth points, so the estimate lines disappear.

As objectives (2) and (4) have closed-form solutions, they motivate a practical bilevel optimization routine described in Alg. 1 and visualized in Figure 1. Note that the unit projection does not affect the permutation in the next iteration, so we omit it until the vector is returned. We avoid storing both the incidence matrix and intermediate distance vector by using implicit matrix multiplication and a problem-specific matching algorithm Alg. 2. The runtime of the optimization inner loop is derived in Proposition 2. In the same proposition, we also derive a memory efficient implementation that avoids storing intermediate values during optimization.

**Lemma 2.** The priority queue in Alg. 2 uses $z^{\left(t\right)}$ interval order, i.e., $\left(i,\;j)\leq (i^{\prime },\;j^{\prime })\Leftrightarrow (z^{\left(t\right)}[\;j]-z^{\left(t\right)}[i])\leq (z^{\left(t\right)}[\;j^{\prime }]-z^{\left(t\right)}[i^{\prime }]\right)$. This ordering satisfies $i\leq i^{\prime },\;j^{\prime }\leq j$ and implies $\left(i,j)\leq (i^{\prime },j^{\prime }\right)$ when $z^{\left(t\right)}$ is sorted.

**Proof:** Suppose $i\leq i^{\prime }$ and $j^{\prime }\leq j$. It follows sortedness that $z^{\left(t\right)}[i]\leq z^{\left(t\right)}[i^{\prime }]$ and $z^{\left(t\right)}[\;j^{\prime }]\leq z^{\left(t\right)}[\;j]$. This means $z^{\left(t\right)}[\;j^{\prime }]-z^{\left(t\right)}[i^{\prime }]\leq z^{\left(t\right)}[\;j]-z^{\left(t\right)}[i]$, which implies $\left(i^{\prime },j^{\prime })\leq (i,j\right)$. □

**Proposition 2.** Upon termination of Alg. 2, the ouput vector $z^{\left(t+1\right)}$ equals $Q^{⊤}\Pi_{Qz^{\left(t\right)}}^{⊤}D$. This algorithm runs in $O(n^{2}\;\log\;n)$ time and uses O(n) nonnegative integers of non-constant storage, where n is the number of points.

**Algorithm 2:** $Q^{⊤}$ applied to matched *D***Input:**   *m*-Distance vector *D*; *n*-Point vectors $z^{\left(t\right)}$, $z^{\left(t+1\right)}$1: $z^{\left(t+1\right)}[:]\leftarrow 0$ ▷ Zero out the vector2: $z^{\left(t\right)}\leftarrow \text{sort}(z^{\left(t\right)})$ ▷ Sort the incoming point vector3:   frontier ← Min-Interval-Priority-Queue $\left(n,z^{\left(t\right)}\right)$ ▷ $z^{\left(t\right)}$ interval order, *n* interval allocation4:   **for** i∈[1,…,n−1] **do**5: enqueue(frontier,(i,i+1))6:   **end for**7:   **for** t∈[1,…,m] **do**8: (i,j)← Min-Pop(frontier)9: $z^{\left(t+1\right)}[i]\leftarrow z^{\left(t+1\right)}[i]-D[t]$10: $z^{\left(t+1\right)}[\;j]\leftarrow z^{\left(t+1\right)}[\;j]+D[t]$11:   **if** j<n **the**12: enqueue(frontier,(i,j+1))13:   **end if**14:   **end for** 1:   **procedure** Interval-Compare(*z*) 2:   **return** $\left[(i_{1},\;j_{1}\right)$, $\left(i_{2},\;j_{2})]\mapsto (z[\;j_{1}]-z[i_{1}])\leq (z[\;j_{2}]-z[i_{2}]\right)$ ▷ Interval comparison function based on *z* 3:   **end procedure**

**Proof:** We first prove that the priority queue pops the $t^{\text{th}}$ smallest distance during iteration *t*. To do this, we define the sequences $I^{k}={\left(k,k+t\right)}_{t=1}^{n-1}.$ The sequences $I^{1},\ldots ,I^{n-1}$ partition all $\left(\begin{array}{c} n\\ 2 \end{array}\right)$ intervals. Lemma 2 establishes that these chains are in interval-sorted order. Consequently, Alg. 2 produces the smallest unseen interval at each iteration. This holds because the queue holds the smallest element from each sequence, and we add the next one until each sequence has been exhausted.

During iteration *t*, interval (i,j) is the (possibly non-unique) $t^{\text{th}}$ smallest interval. Thus, the sorting permutation will send the interval (i,j) to index *t*, and its transpose (i.e., inverse) will send index *t* to interval (i,j). This means *D*[*t*] is used as the matched (i,j) distance. When multiplying by $Q^{⊤}$, the (i,j) distance entry contributes only to the $i^{\text{th}}$ and $j^{\text{th}}$ points. Specifically, the (i,j) entry is subtracted from $z^{\left(t+1\right)}[i]$ and added to $z^{\left(t+1\right)}[\;j]$, which is immediately performed in Alg. 2’s loop. Thus, the algorithm terminates with $z^{\left(t+1\right)}=Q^{⊤}P_{t}D$ since (i) the array entries are set to zero and then (ii) the contributing entries are accumulated during the sorting steps.

The algorithm performs O(n log⁡ n) work before the main loop. This loop performs $O(n^{2}\;\log\;n)$ work due to the priority queue, which takes O(log n) time per iteration using standard implementations. This is unaffected by the interval comparison function, as it uses only a constant number of operations. The priority queue uses the only non-constant memory, as it stores O(n) non-negative integers for the intervals. □

**Proposition 3.** The outer loop of Alg. 1 terminates in a finite number of steps. The inner loop runs in time $O(n^{2}\;\log\;n)$ and uses O(n) non-constant storage space.

**Proof:** For the first claim, note that a fixed permutation fully decides the updated point vector values. This means a finite number of point vectors can be produced for a specific distance set. Proposition 1 implies that each update of the point vector strictly improves the objective until the algorithm converges. The algorithm cannot strictly improve the objective indefinitely, as finitely many states exist; thus, the procedure terminates when $z^{\left(t\right)}=z^{\left(t+1\right)}$.

For the second claim, notice that steps 5 and 6 of Alg. 1 take $O(n^{2}\;\log\;n)$ time and need only O(n) non-negative integers of non-constant storage by the result of Proposition 2. We also only need to keep two *n* floating point vectors to calculate step 8. □

In practice, we observed that Alg. 1 converges quickly but is prone to becoming trapped in local maxima. To prevent this, we further propose a divide-and-conquer heuristic formally described in Alg. 3. In particular, after each pass of Alg. 1, we partition the estimation $\hat{z}$ into non-overlapping subsets ${\hat{z}}_{l}$ and ${\hat{z}}_{r}$. In our implementation, the median is used to form the partitions, but any rule works with this framework. No matter the choice, this segments the distance set *D* into three portions: (a) $D_{ll}$ contains the distances among points in ${\hat{z}}_{l}$; (b) $D_{rr}$ contains the distances among points in ${\hat{z}}_{r}$; and (c) $D_{lr}$ contains the distances between pairs of points respectively in ${\hat{z}}_{l}$ and ${\hat{z}}_{r}$. Even though we do not have the ground truth assignment of the point-distance correspondence, we can use the estimated permutation matrix *P* to perform this segmentation.

**FIG. 2.**
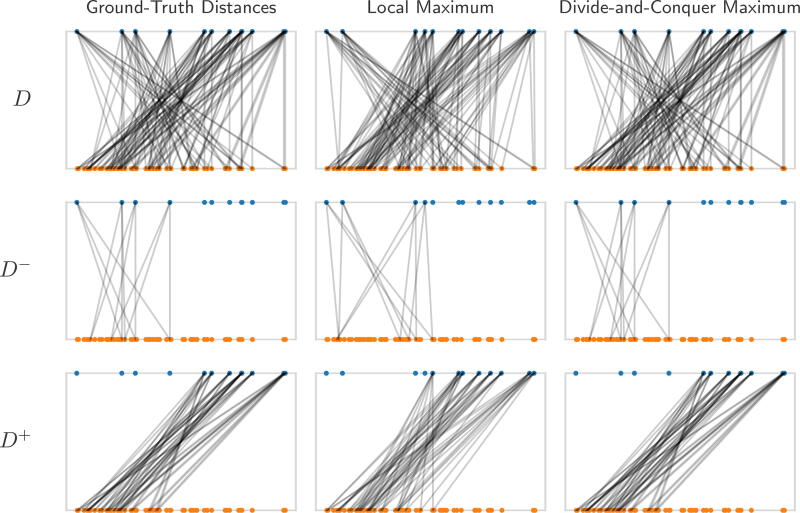
An example of the divide-and-conquer strategy applied to a local maximum, leading to global optimality. The figure presents a series of plots, where the point vector (*blue*) at the top corresponds to the distance vector (*orange*) at the bottom. Each distance has two edges to the points that generated it. From top to bottom, the rows represent the full distance set, the distance set associated with negative points, and the distance set associated with non-negative points. From left to right, we see the ground truth matching, local maximum matching, and divide-and-conquer maximum matching. This instance displays the ideal case for the divide-and-conquer approach, where each half of the interval gets nearly its entire distance set.

The intuition behind our proposed heuristic is that, if $\hat{z}$ and *P* are the optimal Turnpike solution, then subsequent applications of Alg. 1 on $\left(z_{l},D_{ll}\right)$ and $\left(z_{r},D_{rr}\right)$ will not alter this solution. Otherwise, the recursive sub-routines will likely not get trapped in the same local maxima as the parent routine and will serve as a self-correcting mechanism for Alg. 1 by returning the adjusted permutations $P_{l}$ and $P_{r}$. At this point, we can adjust ${\hat{z}}_{l}$ and ${\hat{z}}_{r}$ by solving the following regression tasks:

$${\hat{z}}_{l}^{+}\;=\;\underset{{z\prime }_{l}}{\text{argmin}}\;\left\|Q_{l}{z\prime }_{l}-P_{l}D_{ll}\right\|\mathrm{and}\;{\hat{z}}_{r}^{+}\;=\;\underset{{z\prime }_{r}}{\text{argmin}}\;\left\|Q_{r}{z\prime }_{r}-P_{r}D_{rr}\right\|,$$

where $Q_{l}$ and $Q_{r}$ are the respective incidence submatrices corresponding to ${\hat{z}}_{l}$ and ${\hat{z}}_{r}$. To avoid storing the incidence matrices, we can use any matrix-free solver such as the conjugate gradient method (Wendland, 2017) along with the matrix-free oracles given in Alg. 4. As the adjusted estimation ${\hat{z}}^{+}=({\hat{z}}_{l}^{+},{\hat{z}}_{r}^{+})$ breaks away from potential local maxima, this routine is repeated until convergence, as described in Alg. 3. We provide a visualization of how this improves solutions in Figure 2.

**Algorithm 3:**   MM Divide-and-Conquer (MMDQ)**Input:  ** Distance vector *D*, initial estimate $z^{\left(0\right)}$, tolerance ϵ1: $D\leftarrow D^{↑}$ ▷ Replace *D* with its sorted equivalent $D^{↑}$2: t←03:   **while not** converged **do**4: $z^{\left(t+1\right)},P_{t+1}\leftarrow \mathrm{MM}(D,z^{\left(t\right)},\epsilon )$ ▷ Alg. 15: $z_{l},z_{r}\leftarrow \mathrm{Partition}(z^{\left(t+1\right)})$ ▷ as described above6: $D_{ll},D_{rr},D_{lr}\leftarrow \mathrm{Segment}(z_{l},z_{r},D,P_{t+1})$ ▷ as above7: $P_{l},\_\leftarrow \mathrm{MMDQ}(D_{ll},z_{l},\epsilon )$ ▷ recursive call on left set8: $P_{r},\_\leftarrow \mathrm{MMDQ}(D_{rr},z_{r},\epsilon )$ ▷ recursive call on right set9: $z^{\left(t+1\right)}\leftarrow$ solve Eq. (2.2) ▷ “consensus” point set10: t←t+111:   converged $\leftarrow ∥z^{\left(t+1\right)}-z^{\left(t\right)}∥<\epsilon$12:   **end while**13:   **return** $P_{t},z^{\left(t\right)}$

**Algorithm 4:** Q and $Q^{⊤}$ multiplication oracles1:   **procedure** *Qz*  (*d*, *z*)2: k←13:   **for** width∈[1, n) **do** ▷ Bottom-up distance indexing4:   **for** i∈[1, n−width] **do**5: d[k]←z[i+width]−z[i]6: k←k+17:   **end for**8:   **end for**9:   **end procedure**1:   **procedure** $Q^{⊤}d$  (*d*, *z*)2: k←13: z[:]←0 ▷ Zero out the vector4:   **for** width∈[1, n) **do** ▷ Bottom-up distance indexing5:   **for** i∈[1, n−width] **do**6: j←i+width7: z[i]←z[i]−d[k]8: z[ j]←z[ j]+d[k]9: k←k+110:   **end for**11:   **end for**12:   **end procedure**

**Remark.** An alternative approach (which is compared against our method in Section 3) to solving Eq. (1) applies Birkhoff’s theorem (Birkhoff, 1946), which states that the polytope $B_{m}$ of m×m doubly stochastic matrices is the convex hull of $S_{m}$. This motivates a relaxation of Eq. (1) to optimize for *P* on $B_{m}$:

(5) $$\hat{z}=\underset{z\prime \;\in \;Z}{\text{argmin}}\;\underset{P\;\in \;B_{m}}{\text{max}}\;\langle Qz\prime ,PD\rangle ,$$

allowing for a differentiable permutation learning framework that combines (a) stochastic gradient descent over the space of square matrices; and (b) projection onto $B_{m}$ with the Sinkhorn operator (Mena et al., 2018). In the case of the Turnpike and Noisy Turnpike problems, this approach requires the algorithm to optimize an infeasibly large m×m matrix. We refer to this alternative as the “gradient descent” method in the results below.

### 2.3. Beltway formulation

In the Beltway problem, we are given n(n−1) unlabeled arc lengths between *n* points $p_{1},\ldots ,p_{n}$ on a circle, where distance refers to the arc length between points. Note that we receive double the number of distances as in the turnpike case because there are two different arcs between any two points (i.e., clockwise and counter-clockwise). For convenience, we only consider problem instances on a unit circle as we are able to scale the points using the transformation detailed in Huang and Dokmanić (2021).

The Beltway problem can be solved within our framework (Section 2.2) with some minor modifications. In particular, we will solve for the angle vector $\vec{\theta }\in {\left[-2\pi ,2\pi \right]}^{n}$ because the arc length between two points on the unit circle is simply the angle between them. This has precisely the same symmetry-breaking constraints as a linear point vector. We constrain the points to this set in order to cover the circle twice using two different orientations. With this setup, the existence of any solution guarantees that there exists at least one solution with an angle vector $\vec{\theta }\in {\mathbb{R}}^{n}$ where $\theta_{1}=0$ because a rigid rotation does not change the pairwise distances. Similar to the turnpike case, we can also freely reorder the angles without changing the problem, so we constrain our search to a sorted angle vector $\theta_{1}\leq \theta_{2}\leq \cdot \cdot \cdot \leq \theta_{n}$.

We account for the two different arcs between any two points by solving for a *2n* dimensional angle vector indexed by integers in [−n,n]−{0}. Let this new vector be defined as the concatenation $\theta^{+}=[\;\vec{\theta }-2\pi |\vec{\theta }\;]$, which constrains our search by the relationship between the clockwise and counter-clockwise arcs. This fixes the ambiguity problem, as both arclength distances are in the standard linear distance set of this vector. To see this, we have for i<j that $\left(\theta_{j}^{+}-\theta_{i}^{+}\right)+\left(\theta_{i}^{+}-\theta_{n-j}^{+}\right)=\vec{\theta_{j}}-\vec{\theta_{i}}+\vec{\theta_{i}}-(\vec{\theta_{j}}-2\pi )=2\pi$, i.e., the entire partition of the circle is present. We also maintain sorted order because $\vec{\theta }$ is sorted, $\vec{\theta }-2\pi$ is sorted, and $\vec{\theta }\geq 0$. Every distance is duplicated because every point has two representatives, so we have to duplicate the distance set before running the algorithm. There are also *n* distances of the form $\theta_{k}-\theta_{-n+k}$ missing, but these are simply the circumference of the circle, i.e., 2π. Alg. 2 can be modified to correctly assign these extra distances with a single conditional.

### 2.4. Extension to problem variants

#### 2.4.1. Motivation

The labeled partial digest problem (LPDP) (Pandurangan and Ramesh, 2002) is a variant of the Turnpike problem where we receive both the endpoint distances $E=\cup_{j=2}^{n-1}\left\{z_{n}-z_{j},z_{j}-z_{1}\right\}$ and the all-pairs distance set *D*. The simplified partial digest problem (SPDP) (Blazewicz et al., 2007) provides an adjacent distance set $A=\cup_{j=1}^{n-1}\left\{z_{j+1}-z_{j}\right\}$ and the same endpoint distance set *E*. For LPDP, we could use the base procedure of Alg. 1, but that does not make efficient use of all available information. For SPDP, we could use a construction similar to the Beltway formulation given in Section 2.3, but that would also not efficiently use the additional labeling information. To that end, we now abstract both problems into a unified framework that builds off of our base algorithm via distance set partitions. To use this framework for LPDP, we require a preprocessing step where we turn the input into a partition of *D*. This can be done by replacing *D* with D−E. In the uncertain case, we can approximate D−E optimally with respect to the $\ell_{1}$ norm between the assigned points in O(n log⁡ n) time using the pool-adjacent-violators algorithm (Lim and Wright, 2016). To form a partition for SPDP, we need to (1) remove the two overlapping distances between *A* and *E*, i.e., $\left(z_{n}-z_{n-1}\right)$ and $\left(z_{2}-z_{1}\right)$, and (2) simulate the missing distances D−(A∪E) with that step’s estimated point vector.

#### 2.4.2. Partition formulation

Suppose we receive as input a partitioned distance set $D=D^{\left(1\right)}⊔\cdot \cdot \cdot ⊔\;D^{\left(k\right)}$ and a partition of linear indices $I^{\left(1\right)}⊔\cdot \cdot \cdot ⊔I^{\left(s\right)}=\{1,\ldots ,m\}=:[m]$ such that the submatrix $Q^{\left(\;j\right)}$ contains the measurements that generated the distance subset $D^{\left(\;j\right)}$. (Stated differently, $Q^{\left(\;j\right)}$ is the incidence matrix of the subgraph induced by the edges corresponding to the distance measurements.) For each $D^{\left(\;j\right)}$, we optimize a $\left|I^{\left(\;j\right)}\right|$ permutation matrix $P^{\left(\;j\right)}$ that rearranges $D^{\left(\;j\right)}$ in the maximizing order. Rather than sorting the combined distance vector *D*, we individually sort each component $D^{\left(\;j\right)}$ in advance. Our reformulated objective becomes

$$\underset{z,\;P^{\left(1\right)},\;\ldots ,\;P^{\left(k\right)}}{\max}\;\sum_{j\;\;⩽\;\;k}\;\langle P^{\left(\;j\right)}D^{\left(\;j\right)},Q^{\left(\;j\right)}z\rangle .$$

As before, the point vector *z* is centered and sorted. Suppose that we fix all the permutation matrices except $P^{\left(\;j\right)}$. Then the terms that do not involve $P^{\left(\;j\right)}$ are constant and can be dropped without loss of optimality. After removal of these terms, the objective is simplified to

$$\underset{P^{\left(\;j\right)}}{\max}\langle P^{\left(\;j\right)}D^{\left(\;j\right)},Q^{\left(\;j\right)}z\rangle .$$

From Lemma 1 and Proposition 1, an optimal choice of $P^{\left(\;j\right)}$ is the transposed sorting permutation for $Q^{\left(\;j\right)}z$. On the other hand, when the permutation matrices are fixed and *z* is not, the objective reduces to the original:

$$\underset{z}{\max}\sum_{j\;\;⩽\;\;k}\langle P^{\left(\;j\right)}D^{\left(\;j\right)},Q^{\left(\;j\right)}z\rangle \equiv \underset{z}{\max}\langle \left(P^{\left(1\right)}+\cdot \cdot \cdot +P^{\left(\;j\right)}\right)\;D,Qz\rangle .$$

Note that this is a slight abuse of notation, since we cannot add the permutation matrices; however, this is fine because we can simply lift each permutation to a partial permutation with zeros on all disjoint indices. Thus, an optimal choice is given by the same closed form presented in Alg. 1, i.e., $z=\text{unit}\left[Q^{⊤}(P^{\left(1\right)}+\cdot \cdot \cdot +P^{\left(k\right)})D\right]$.

#### 2.4.3. Missing distances

Suppose distances $D^{\left(0\right)}$ corresponding to an index set $I^{\left(0\right)}$ are missing from the input. Letting $I^{\left(0\right)}$ denote the indices of the missing subset, the index partition is then $[m]=I^{\left(0\right)}⊔\;I^{\left(1\right)}\cdot \cdot \cdot ⊔\;I^{\left(k\right)}$. Our formulation can accommodate for such missing entries with a simple change: we use the current estimate $z^{\left(t\right)}$ to simulate the missing distances when estimating $z^{\left(t+1\right)}$. This results in no change to the other subproblems, as the permutations are estimated only for distances assigned to the respective indices.

#### 2.4.4. Block-update algorithm

We have a closed-form solution to every subproblem, and the objective is nondecreasing with respect to each closed subproblem. Thus, we can use block updates (i.e., update one subproblem at a time) or parallel updates (i.e., update and aggregate all subproblems) to make progress. For simplicity, the version we present (Alg. 5) is an extension of Alg. 1 that updates all permutations in parallel and then updates the point vector. We stop the optimization once the point vector converges and adapt Alg. 2 to run on the partitioned distance set while preserving the O(n) space complexity, provided the partition index of an interval can be queried efficiently (discussed later).

**Algorithm 5:**   Block-Descent Minorization-Maximization (MM)**Input:**  Available distances $D-D^{\left(0\right)}={⊔}_{j=1}^{k}D^{\left(\;j\right)}$, Index Partition $I={⊔}_{j=0}^{k}I^{\left(\;j\right)}$, Initializer $z^{0}$, Tolerance ϵ>01: t←02: $\forall j\in \{1,\ldots ,k\},\;D^{\left(\;j\right)}\leftarrow {\left(D^{\left(\;j\right)}\right)}^{↑}$ ▷ Sort each distance vector in the partition3:   **while not** converged **do**4: $\forall j\in \{1,\ldots ,k\},P_{t}^{\left(\;j\right)}\leftarrow \Pi_{Q^{\left(\;j\right)}[z^{\left(t\right)}]}^{⊤}$ ▷ Calculate a sorting permutation for each $Q_{I_{j}}[z^{\left(t\right)}]$5: $z^{\left(t+1\right)}\leftarrow Q^{⊤}\left(Q^{\left(0\right)}z^{\left(t\right)}+\left(\;P_{t}^{\left(1\right)}+\cdot \cdot \cdot +P_{t}^{\left(k\right)}\;\right)D\right)$ ▷ Estimate the next point vector, fill in missing distances6: t←t+17:   converged $\leftarrow ∥z^{\left(t+1\right)}-z^{\left(t\right)}{∥}_{2}<\epsilon$8:   **end while**9:   **return** $\text{unit}(z^{\left(t\right)})$

**Algorithm 6:** $Q^{⊤}$ applied to matched *D* with a partition**Input:**   Available distances $D-D^{\left(0\right)}={⊔}_{j=1}^{k}D^{\left(\;j\right)}$; *n*-Point vectors $z^{\left(t\right)}$, $z^{\left(t+1\right)}$1: $z^{\left(t+1\right)}[:]\leftarrow 0$ ▷ Zero out the vector2: $z^{\left(t\right)}\leftarrow \text{sort}(z^{\left(t\right)})$ ▷ Sort the incoming point vector3:   frontier ← Min-Interval-Priority-Queue $\left(n,z^{\left(t\right)}\right)$ ▷ $z^{\left(t\right)}$ interval order, *n* interval allocation4:   **for** i∈[1,…,n−1] **do**5: enqueue(frontier,(i,i+1))6:   **end for**7: $\forall j\in \{1,\ldots ,k\},\;t^{\left(\;j\right)}=1$ ▷ Initialize partition indices8:   **for** t∈[1,…,m] **do**9: (i,j)← Min-Pop(frontier)10: p← partition-index(i,j) ▷ Find *p* such that $(i,j)\in I^{p}$11:   **if** p=0 **then**12: $d\leftarrow z^{\left(t\right)}[\;j]-z^{\left(t\right)}[i]$ ▷ Fill in missing distance13:   **end if**14:   **if** p>0 **then**15: $d\leftarrow D^{\left(p\right)}\left[t^{\left(p\right)}\right]$16: $t^{\left(p\right)}\leftarrow t^{\left(p\right)}+1$17:   **end if**18: $z^{\left(t+1\right)}[i]\leftarrow z^{\left(t+1\right)}\left[i\right]-d$19: $z^{\left(t+1\right)}[\;j]\leftarrow z^{\left(t+1\right)}\left[j\right]+d$20:   **if** j<n **then**21: enqueue(frontier,(i, j+1))22:   **end if**23:   **end for**

There are three primary differences between Alg. 3 and Alg. 6.

1. Before the loop, we initialize indices $t^{\left(1\right)},\ldots ,t^{\left(k\right)}$ for the sets in the provided partition.
2. When a missing distance is relaxed, we simulate the distance using $z^{\left(t\right)}$.
3. When an interval in $I^{\left(\;j\right)}$ with j≠0 is relaxed, we use the distance $D^{\left(\;j\right)}[t^{\left(\;j\right)}]$ and set $t^{\left(\;j\right)}\leftarrow t^{\left(\;j\right)}+1$.

These changes ensure a distance from the correct partition segment is assigned to the current interval, and if it is missing, it is filled in from the current vector $z^{\left(t\right)}$. Note that the asymptotic runtime is unaffected, aside from the cost of checking the interval’s partition index. This step is run once per iteration, so the runtime becomes $O(n^{2}\;\log\;n+pn^{2})$, where *p* is the worst-case cost of checking the partition index. For natural partitions, checking the index takes constant time and requires no additional space, leaving both runtime and space complexity unchanged. For example, in LPDP, we only check whether the current interval has endpoint 1 or *n*, and in SPDP, we check if the interval is from two adjacent points, if it is from an endpoint distance, or if it is from neither.

### 2.5. Initializer sampling

The choice of an initializer for Algs. 3 and 5 is critical to both convergence and reconstruction quality, as a well-chosen initializer leads to rapid convergence, improved stability, and a more accurate solution. Here, we consider three practical initializing schemes. The first scheme samples a random Gaussian vector and sorts it. Though efficient to implement, this scheme is unlikely to produce a good initializer if the ground set exhibits pathological features such as having spread-out point clusters. On the other hand, if the points are well-spread, this scheme often finds a close starting point. The second scheme provides a random permutation $P_{0}$ to the sub-problem in Eq. (2) and sets $z^{\left(0\right)}$ as its closed-form solution. This incorporates the combinatorial nature of Turnpike and potentially encourages more diverse exploration of the solution space. Nevertheless, selecting random permutations does not guarantee proximity to the optimal solution or even proximity to a valid distance permutation. The final scheme is a greedy-search method inspired by the classical backtracking approach (Skiena and Sundaram, 1993). That is, we sequentially fit the largest distance in *D* onto a line segment configuration (i.e., placing a new point to the left or to the right end of the segment based on this distance). However, unlike the original formulation—which uses backtracking to find the optimal placement—we make greedy choices to generate an initializer that will be polished with our algorithm afterwards, thus avoiding potentially exponential runtime.

### 2.6. Integer programming formulations

In this section, we develop an integer programming analog of Alg. 5 in order to provide global optimality certificates. To efficiently model our problems, we do not explicitly represent the permutation matrix of Problem 1, as that requires $\Theta (n^{4})$ binary variables. Instead, we implicitly store the permutation as a vector of sorted variables, which we implement with Goemans’ extended formulation (Goemans, 2015) using only $\Theta (n^{2}\;\log\;n)$ binary variables, continuous variables, and linear constraints. (Though, we need $\Theta (n^{2}\;\log^{2}\;n)$ constraints and variables in practice, as the Θ(·) notation suppresses a rather large constant in the idealized construction.) To capture all problem variants, we develop the generic constraint set below.

**Lemma 3**. Let *m* and $t_{1},\ldots ,t_{k}$ be natural numbers such that $t_{1}+\cdot \cdot \cdot +t_{k}=m$, and suppose that ${\left\{T^{\left(\;j\right)}\right\}}_{j=0}^{k}$ are linear maps such that $T^{\left(\;j\right)}:{\mathbb{R}}^{n}\to {\mathbb{R}}^{t_{j}}$. The following constraints are representable with O(m log⁡ m) binary variables, O(m log⁡ m) continuous variables, and O(m log⁡ m) linear constraints.

$$\begin{array}{l} \;x\in {\mathbb{Q}}^{n},\\ \;\forall j\in [0,k],\;y^{\left(\;j\right)}\in {\mathbb{Q}}^{t_{j}},\\ \;\forall j\in [0,k],\;y^{\left(\;j\right)}=\text{sort}(T^{\left(\;j\right)}x). \end{array}$$

**Proof:** The stated bounds clearly hold for all but the sorting constraints, and the sorted vectors can be represented by Goemans’ extended formulation, as discussed above. For all sorting constraints, this totals to $\sum_{j=1}^{k}\Theta (t_{j}\;\log\;t_{j})\in O(m\;\log\;m)$ since $t_{1}+\cdot \cdot \cdot +t_{k}=m$. □

Recall that a (strong) separation oracle for a convex set A takes a point *x* as input and returns a hyperplane separating *x* from the set when *x* is not in A. In integer programs, such oracles provide *lazy constraints* that activate only as required to cut invalid solutions. We use separation oracles to transform our bilinear distance-matching formulation into an integer linear program. We do this with a *support function*, i.e., a function $\sigma_{X}(z)=\max_{x\;\;\in \;\;X}\;\langle x,z\rangle$ for a fixed set of vectors X. Such functions are convenient because, when the supporting set is finite, minimizing them is equivalent to an (integer) linear program with constraints provided by a separation oracle (see Theorem 1 for details). In our case, this is useful because Objective 2.4 is a support function for a large yet finite supporting set, where Alg. 5 implements the desired separation oracle. As an added benefit, this choice provides the freedom to consider general support function objectives, which opens a wide class of distance-like convex functions that includes norms. [For further details, see the support function chapter of Bauschke and Combettes (2019).] This leads to the following integer linear programming transformation of the distance matching problem introduced in Section 2.4.

**Theorem 1.** For $m=\left(\begin{array}{c} n\\ 2 \end{array}\right)$, suppose $I^{1}\;⊔\;\ldots \;⊔\;I^{k}=[m]$ is an index partition with sizes $m_{1}+\cdot \cdot \cdot +m_{k}=m$ such that $Q^{j}$ is the incidence matrix of the subgraph induced by the indices in $I^{j}$. Let $D^{1},\ldots ,D^{k}$ be distance sets in sorted order and $\sigma_{X}:({\mathbb{R}}^{m_{1}},\ldots ,{\mathbb{R}}^{m_{k}})\to \mathbb{R}$ be a support function of a finite, rational supporting set X. The following program is equivalent to an integer linear program with lazy constraints enforced by a separation oracle, $O(n^{2}\;\log\;n)$ binary variables, $O(n^{2}\;\log\;n)$ continuous variables, and $O(n^{2}\;\log\;n)$ linear constraints.

$$\begin{array}{ll} \min\;\;\sigma (r^{\left(1\right)},\ldots ,r^{\left(k\right)})\\ \mathrm{subject}\;\mathrm{to}\;z\in {\mathbb{Q}}^{n}, & \\ \;\forall j\in [k],r^{\left(\;j\right)}\in {\mathbb{Q}}^{\left(m_{j}\right)}, & \\ \;\forall j\in [k],D^{\left(\;j\right)}=r^{\left(\;j\right)}+\text{sort}(Q^{\left(\;j\right)}z). & \end{array}$$

**Proof:** Lemma 3 implies that the stated bounds hold for these constraints. The objective can be modeled as the minimization of an auxiliary variable t∈ℚ such that

$$t\;⩾\;\underset{x\;\;\in \;\;X}{\max}\;\langle x,\left(r^{\left(1\right)},\ldots ,r^{\left(k\right)}\right)\rangle ,$$

where the separation oracle produces a vector in X realizing a higher value if higher than *t*. As *t* is minimized, the inequality becomes an equality at optimality. □

In this formulation, the residual vectors $r^{1},\ldots ,r^{k}$ represent the failure of the input distances to match the distances generated by *z*. To implement the objective, these residuals are scored by the specified support function. In Section 6, we explore three possible objectives that are compared in terms of runtime and reconstruction quality.

## 3. EMPIRICAL RESULTS

### 3.1. Experimental design

We assessed the performance of our proposed algorithm on synthetic data as a baseline and compared its performance to the backtracking method (Skiena and Sundaram, 1993), the distribution matching method (Huang and Dokmanić, 2021), and our projected gradient descent baseline using the Gumbel-Sinkhorn relaxation (Mena et al., 2018). To evaluate the performance of our method in genome reconstruction, we conduct a series of experiments that aim to reconstruct a DNA sequence from fragments generated by synthetic enzymes. All experiments were implemented in Python 3.10 using a C++20 library implementing the algorithm integrated with Python using PyBind11, and conducted on a computer equipped with 1.0 TB of RAM and two Intel Xeon E5-2699A v4 CPUs.

### 3.2. Synthetic data

Synthetic datasets were generated by sampling *n* points on the real line from three distributions: the Cauchy distribution, the standard normal distribution, and the uniform distribution on *[0, 1]*. The uniform distribution was chosen to align with the setting explored by Huang and Dokmanić (2021). The normal distribution was chosen to test point sets with clustered points. The Cauchy distribution was selected to test the effect of contrasting scales, and dispersion of points sampled from the Cauchy distribution presents a challenge for $\ell_{2}$ optimization methods, which struggle with outlying values (Boyd et al., 2004).

**FIG. 3.**
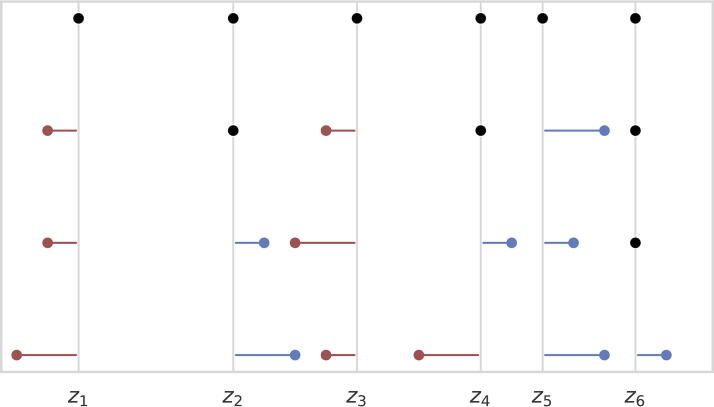
From top-to-bottom, we see the ground truth point set and then point sets that are an MAE of $10^{-4}$, $10^{-3}$, and $10^{-2}$ away from the ground truth. MAE, mean absolute error.

We examined sample sizes ranging from 50 to 2000 points (in increments of 50) and three additional large sample sizes of 5000, 10,000, and 100,000 to demonstrate the method’s scalability. To simulate measurement uncertainty of magnitude $\epsilon =10^{-k}$ for integer k∈[1,7], we added a Gaussian noise vector g∼N(0,ϵI) to the given vector of pairwise distances (Dokmanic et al., 2015). We rounded the distance to zero when the amount of uncertainty exceeded the magnitude of the distance, which simulates missing distances. We predicted the points for each set of distribution, size, and uncertainty for 10 independent test cases. We ran each algorithm 10 times and output the best estimate, which we quantified with the $\ell_{2}$ distance between the estimated and uncertain distance sets (the algorithms are deterministic, but the choice of initializer is random as described above). We recorded the mean absolute error (MAE) and mean squared error (MSE) between the estimated and ground point sets (see Fig. 3 for a visualization). The MAE is a continuous alternative to the binning distance (Huang and Dokmanić, 2021) and is more suitable for our method since we do not explicitly assign points to bins. Since the distribution matching method produces bins as its output, we use the midpoint of each bin as the predicted point.

### 3.3. Study of different initialization schemes

**FIG. 4.**
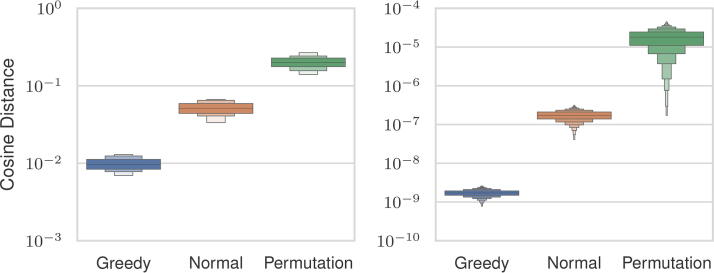
Cosine similarities between estimated and ground distance vectors ( $\hat{D}$ and *D*) before (*left*) and after (*right*) MM optimization for three different initialization schemes. MM, Minorization-Maximization.

We investigated the three initialization strategies (Section 2.5) to select one for subsequent experiments. We boosted the Gaussian point vector and permutation point vector initializer by drawing *n* distinct samples that were scored by solving Problem 2 for each and taking the maximum value. The sample with the maximum score from each strategy was used as the initializer. We used the Gaussian initializer as the starting point for the greedy-search initializer. We tested the strategies across all settings described previously. Figure 4 shows the cosine distances between the estimated and uncertain distance vectors. Among the three approaches, the permutation strategy exhibited the worst similarity scores, with an average magnitude 13 times larger than that of the greedy-search strategy. The Gaussian strategy demonstrated an average error magnitude that was 8 times larger than the greedy-search initialization.

A better initial score does not necessarily guarantee a better reconstruction after optimization. To assess the efficacy of each initializer, we analyzed whether lower pre-optimization errors translated to reduced post-optimization errors. The cosine distance after optimization is also shown in Figure 4. The permutation initialization had the highest errors, and the greedy-search approach had the lowest errors, which is consistent with the pre-optimization cosine distance. We used the greedy-search initializer for our experiments since it exhibited the lowest post-optimization distance. The greedy-search strategy’s lower error comes at a computational cost. Table 1 shows the median runtime for the greedy-search initializer, Gaussian initializer, and optimization loop across a representative set of problem sizes. For all sizes, the greedy-search initialization takes more time than running the optimization, whereas the Gaussian initialization strategy runs in an order of magnitude less time than the optimization. This is due to the inherently serial nature of the greedy-search initializer, which requires all previous steps to be considered first. This is in contrast to the optimization loop, which has a runtime dominated by sorting, which is parallelized.

**Table 1.** Median Runtimes (in Seconds) for the Minorization-Maximization Optimizer, Gaussian Initializer, and Greedy Initializer over Different Sample Sizes

Points	100	500	1000	1500	2000
Optimizer	0.40	8.40	16.49	25.39	42.39
Gaussian	0.54	2.54	4.63	7.53	18.53
Greedy	0.23	17.56	36.49	55.72	84.06

### 3.4. Accuracy and robustness of Noisy Turnpike solutions on synthetic instances

We tested how accurately the MM (Section 2.2), backtracking, distribution matching (Huang and Dokmanić, 2021), and gradient descent methods were able to reconstruct point sets. Table 2 shows the median MAE normalized by the uncertainty for a representative set of problem sizes and uncertainties. Each method had 1 hour to solve each instance, with the exception of 10,000 and 100,000 point instances, which were given 90 and 6000 minutes, respectively. The backtracking method was able to solve instances with 1,000 or fewer points, but exhibited larger errors than our method. The gradient descent method solved all instances with 500 or fewer points with residual error that ranged between 10 and 1,000 times higher than the MM approach. The distribution matching method performed similarly to our method but could not scale past 100 points. Our method was able to solve instances with 2,000 points with a median MAE that is 10 times lower than the uncertainty level up to a magnitude of $10^{-4}$, after which the scaling becomes distribution dependent.

**Table 2.** Median Mean Absolute Error Normalized by the Magnitude of Measurement Uncertainty ϵ across Different Point Set Sizes and Uncertainties

	$10^{-6}$				$10^{-5}$				$10^{-4}$			
*n*	MM	DM	BT	GD	MM	DM	BT	GD	MM	DM	BT	GD
100	0.2	0.49	26.4	1270	0.19	0.51	26.6	186	2.09	0.68	56.7	1.54
200	0.1	—	26.4	462	0.14	—	26.5	39	1.54	—	55.7	5.72
500	0.11	—	36.4	312	0.09	—	31.5	40	0.94	—	34.7	3.08
1000	0.06	—	36.4	—	0.04	—	36.5	—	0.08	—	36.7	—
5000	0.08	—	—	—	0.08	—	—	—	0.86	—	—	—
10000	0.08	—	—	—	0.11	—	—	—	0.82	—	—	—
100000	0.12	—	—	—	0.08	—	—	—	0.11	—	—	—

We compare our method (Minorization-Maximization, MM), Distribution Matching (DM), Backtracking (BT), and Gradient Descent (GD) Approaches. A dash indicates that a method did not finish solving any instances of this size due to either memory or runtime constraints.

**FIG. 5.**
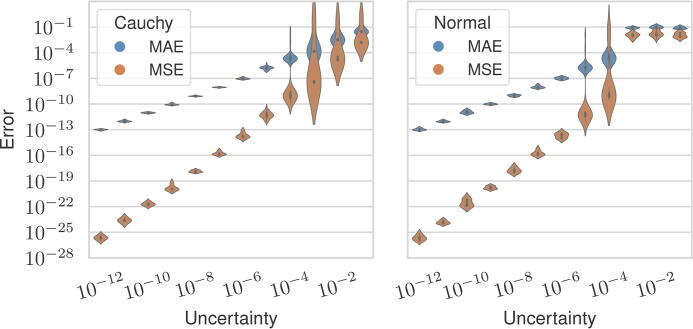
Mean absolute error (*blue*) and mean squared error (*orange*) between the estimated and ground vectors across all levels of measurement uncertainty and separated by distribution.

Figure 5 shows our method’s MAE and MSE over all settings plotted with respect to the magnitude of uncertainty. We observed that uncertainty in the distances correlated with reconstruction error, but the MAE is an order of magnitude lower than the uncertainty on average when the uncertainty is $10^{-4}$ or less. Instance size also affects the method’s error scaling. As the sample size varied between 50 and 100,000 points, the median MAE shown in Table 2 demonstrates a downward trend for fixed error rates. This suggests the method scales at least as well as it does on small point sets as the number of points grows. This is because the distance measurement linear system is highly overdetermined, which makes it resilient to uncertainty (Needell, 2010). Last, we report the mean and standard deviation of the runtimes of different solvers in Table 3. The MM mean runtime was lowest across all point sizes, and MM is the only method that successfully solved the 5,000, 10,000, and 100,000 point instances.

**Table 3.** Mean Runtime in Seconds with Standard Deviations across Point Sizes for Various Turnpike Solvers

*n*	MM	DM	BT	GD
100	0.8 ± 3.9	1680.3 ± 32.1	2.8 ± 10.2	16.3 ± 0.4
200	21.2 ± 13.0	—	53. ± 32.3	114.9 ± 0.5
500	204.3 ± 74.4	—	304.9 ± 20.3	4366.6 ± 8.6
1000	552.3 ± 112.4	—	1052.5 ± 50.9	—
5000	1992.1 ± 51.2	—	—	—
10000	3543.8 ± 71.2	—	—	—
100000	5912.3 ± 52.4	—	—	—

BT, Backtracking; DM, Distribution Matching; GD, Gradient Descent; MM, Minorization-Maximization.

### 3.5. Partial digestion experiments

We test the use of our algorithms for reconstructing genomes in the setting that uses an enzyme to partially digest DNA fragments at restriction sites (Alizadeh et al., 1995). The fragment lengths give the distances between all restriction sites, which are at unknown positions. The genome is assembled from the fragments after inferring the restriction site locations from the distances, a process equivalent to solving the Turnpike problem for linear genomes and the Beltway problem for circular genomes (Huang and Dokmanić, 2021). We simulated partial digestion instances to test our algorithms. For Turnpike instances, we used the human X chromosome’s centromere, and for Beltway instances, we used the full genome of the bacteria *Carsonella ruddii*. In both cases, we used 15-base-long enzymes and simulated the digestion process by sampling the DNA sequence such that each restriction site occurred between 10 and 500 times. We obtained digested DNA fragments by splitting the sequence at all of its occurrences. We added a signed Poisson random vector parameterized by a value λ>0. This simulates when the enzymes cut too many or too few bases, both frequent occurrences in practice (Cieliebak and Eidenbenz, 2004). The Turnpike experiments were performed with our method and the gradient descent baseline, as they timed out for the other methods due to the size and amount of uncertainty. The Beltway experiments were performed with our algorithm and the distribution matching algorithm, as they are the only ones designed for uncertain Beltway instances.

Table 4 shows normalized MAE for the Turnpike experiments, which were performed with 10 to 2669 fragments. Our method recovered fragment locations with an MAE that scaled linearly to the uncertainty present in the measurements, performing orders of magnitude better than the gradient descent baseline. Table 5 shows normalized MAE for the Beltway experiments, which were performed with 10 to 54 fragments. Our method performed competitively with the distribution matching approach. In both cases, the generated point sets had highly repetitive distances, as the simulated restriction sites were frequently equidistant from one another, showing our method’s robustness to symmetric distances.

**Table 4.** Normalized Mean Absolute Error for 10 Sizes and 4 Uncertainty Magnitudes Using the Minorization-Maximization (MM) and Gradient Descent (GD) Algorithms on Simulated Partial Digestions of a Linear Genome

*n*	$10^{-7}$		$10^{-6}$		$10^{-5}$		$10^{-4}$	
	MM	GD	MM	GD	MM	GD	MM	GD
10	0.152	$2.31\times 10^{6}$	0.148	$1.54\times 10^{5}$	0.163	$1.70\times 10^{4}$	0.142	$1.34\times 10^{3}$
64	0.213	$1.89\times 10^{5}$	0.219	$6.42\times 10^{4}$	0.220	$1.87\times 10^{3}$	0.232	$4.20\times 10^{2}$
142	0.358	$8.22\times 10^{5}$	0.359	$3.44\times 10^{4}$	0.357	$9.28\times 10^{3}$	0.365	$6.12\times 10^{2}$
183	0.282	$1.28\times 10^{6}$	0.268	$1.75\times 10^{5}$	0.288	$9.37\times 10^{3}$	0.290	$1.34\times 10^{3}$
530	0.071	—	0.070	—	0.080	—	0.066	—
959	0.119	—	0.121	—	0.125	—	0.139	—
1209	0.119	—	0.132	—	0.127	—	0.115	—
1451	0.059	—	0.061	—	0.043	—	0.072	—
2669	0.048	—	0.039	—	0.050	—	0.048	—

A Dash Indicates That the Size Did Not Finish Due to Memory Constraints or Runtime Constraints.

**Table 5.** Normalized Mean Absolute Error for 10 Sizes and 4 Uncertainty Magnitudes Using the Minorization-Maximization (MM) and Distribution Matching (DM) Algorithms on Simulated Partial Digestions of a Circular Genome

*n*	$10^{-7}$		$10^{-6}$		$10^{-5}$		$10^{-4}$	
	MM	DM	MM	DM	MM	DM	MM	DM
10	0.148	0.087	0.159	0.096	0.168	0.145	0.232	0.284
15	0.157	0.072	0.150	0.078	0.140	0.160	0.202	0.274
20	0.214	0.031	0.186	0.032	0.123	0.068	0.224	0.231
38	0.113	0.043	0.128	0.094	0.135	0.112	0.178	0.243
54	0.101	0.053	0.102	0.078	0.186	0.203	0.146	0.581

### 3.6. Labeled partial digestion experiment

Pandurangan and Ramesh (2002) performed a LPDP recovery experiment using the restriction sites of the enzyme HindIII on the bacteriophage λ.

For each distance *d*, they simulated relative uncertainty of order r∈[0,1] by replacing *d* with a uniformly sampled integer in [(1−r) d,(1+r) d]. They varied *r* between 0% and 5% to mimic experimental settings, where 2% to 5% is expected. Each experiment was repeated 100 times and was reported as a success when the recovered distances were within the relative uncertainty of the ground truth set. We repeated this experiment using our base algorithm (MM; without using additional labeling information) and our partition-update formulation given in Alg. 5. The results are shown in Table 6, where we see our method performs competitively without additional labels and further improves when provided with the labels. All instances ran in less than one second across all uncertainties and solvers.

**Table 6.** Recovery Success by % Relative Error for Our Base Solver (MM), Our Partition Solver (PMM), and the LPDP Solver (Pandurangan and Ramesh, 2002)

*r*	MM	PMM	LPDP
0%	100%	100%	100%
1%	99%	99%	98%
2%	96%	97%	96%
3%	95%	96%	94%
4%	92%	94%	91%
5%	89%	92%	87%

LPDP, The labeled partial digest problem; MM, Minorization-Maximization; PPM, Partition Minorization-Maximization.

### 3.7. Integer program experiment

We tested the integer program of Theorem 1 using three objectives inspired by various distance models. The first uses the support function introduced in Eq. 2.4, where we use Alg. 5 to implement the separation oracle. The second is inspired by the relative error model of Pandurangan and Ramesh (2002), where we use the $\ell_{\infty }$ norm inversely weighted by the input distance set. The third considers a weighted $\ell_{\infty }$ norm, where the distance assigned to interval *st* is penalized according to the worst partitioning pair (sk,kt), i.e., $r_{st}=\max_{k\in (s,t)}|d_{st}-d_{sk}+d_{kt}|$. These metrics were tested on point sets ranging in size from 5 to 30 in increments of 5 for both partial digestions and labeled partial digestions. Each experiment was performed on 20 random instances using the sampling methods described in the partial digestion experiments, where base-cutting uncertainty was added with relative magnitude as in LPDP experiment. Runtime information is in Table 7, and reconstruction metrics are shown in Tables 8 and 9. The three objectives performed similarly, with the first and third having slight biases towards standard partial digestions and the second having a slight bias towards labeled partial digestions. As expected, the runtime of this method is prohibitive for instances as small as n=30 since, even with the extended formulation, we require $∼30^{2}\;\log_{2}{\left(30\right)}^{2}\approx 4000$ binary variables for 30 points. This is a substantial improvement over the $∼30^{4}\approx$ 810,000 binary variables required by the doubly stochastic formulation, but it is unrealistic for large point sets. That said, the timing is reasonable for smaller partial digestions and provides high-quality reconstructions at that scale.

**Table 7.** Mean Runtime in Seconds across All Scenarios for Point Sets of Size *n* for Three Support Function Objectives: The Relative-Error Norm $\ell_{\infty }^{d}$, the Minorization-Maximization Support Function $\ell_{2}^{d}$, and the Partitioning Norm $\ell_{\mathrm{ijk}}$

*n*	5	10	15	20	25	30
** ** $\ell_{\infty }^{d}$	2	11	78	583	1233	6183
** ** $\ell_{2}^{d}$	1	10	67	486	1317	6201
** ** $\ell_{\mathrm{ijk}}$	1	8	32	512	1011	5347

**Table 8.** Average Mean Absolute Error of Recovered Points for Partial Digestions across All Objective Functions

*n*	5	10	15	20	25	30
$\ell_{\infty }^{d}$	$1.1\times 10^{-4}$	$1.3\times 10^{-4}$	$1.2\times 10^{-4}$	$1.4\times 10^{-4}$	$1.2\times 10^{-4}$	$1.1\times 10^{-4}$
$\ell_{2}^{d}$	$1.0\times 10^{-4}$	$1.4\times 10^{-4}$	$1.5\times 10^{-4}$	$1.7\times 10^{-4}$	$1.3\times 10^{-4}$	$1.2\times 10^{-4}$
$\ell_{\mathrm{ijk}}$	$1.2\times 10^{-4}$	$1.2\times 10^{-4}$	$1.2\times 10^{-5}$	$1.4\times 10^{-5}$	$1.3\times 10^{-5}$	$1.4\times 10^{-5}$

**Table 9.** Average Mean Absolute Error of Recovered Points for Labeled Partial Digestions across All Objective Functions

*n*	5	10	15	20	25	30
$\ell_{\infty }^{d}$	$1.4\times 10^{-6}$	$1.3\times 10^{-5}$	$1.2\times 10^{-5}$	$1.8\times 10^{-6}$	$1.5\times 10^{-5}$	$1.4\times 10^{-5}$
$\ell_{2}^{d}$	$1.6\times 10^{-5}$	$1.8\times 10^{-5}$	$1.3\times 10^{-4}$	$1.4\times 10^{-5}$	$1.5\times 10^{-6}$	$1.3\times 10^{-6}$
$\ell_{\mathrm{ijk}}$	$1.8\times 10^{-5}$	$1.9\times 10^{-5}$	$1.4\times 10^{-5}$	$1.5\times 10^{-5}$	$1.8\times 10^{-5}$	$1.7\times 10^{-5}$

## 4. CONCLUSION

The Noisy Beltway and Noisy Turnpike problems aim to recover a set of one-dimensional points based on a corrupted set of pairwise distances. These problems find application in biological contexts and serve as a fundamental component in resolving unassigned distance geometry in higher-dimensional spaces. It is also interesting theoretically, since the Exact Turnpike problem is of undetermined complexity (not known to be in P or NP) and the Noisy Turnpike and Noisy Beltway are known to be NP hard. To address both problems, we introduced a novel optimization formulation for Noisy Turnpike and an alternating algorithm built from sorting and implicit matrix multiplication. This leads to an asymptotic runtime of $O(n^{2}\;\log\;n)$ time per iteration with O(n) auxiliary memory. To escape low-quality local optima, we introduced a divide-and-conquer step to fix common errors. We extended the method to include the Noisy Beltway problem and variants of the Turnpike problem. In addition, we derived and tested integer programming extensions of our methods. We performed large-scale experiments with approximately 25 billion distances (equivalent to 100,000 points) to demonstrate the efficiency of our method. In contrast, previous methods are infeasible with as few as 125,000 distances (equivalent to 500 points). We also demonstrated the robustness and scalability of the method in a variety of challenging situations, including large-scale uncertainty and distance duplication. Our algorithm allows for efficient, scalable solutions to the Turnpike problem that can accommodate large distance sets with realistic levels of uncertainty, opening up new avenues of research into its applications. Moreover, our algorithm can serve as an efficient, low-cost primitive for solving higher-dimensional distance geometry problems.
